# *Inverse association of falciparum* positivity with endemic Burkitt lymphoma is robust in analyses adjusting for pre-enrollment malaria in the EMBLEM case-control study

**DOI:** 10.1186/s13027-021-00377-0

**Published:** 2021-06-07

**Authors:** Sally Peprah, Martin D. Ogwang, Patrick Kerchan, Steven J. Reynolds, Constance N. Tenge, Pamela A. Were, Robert T. Kuremu, Walter N. Wekesa, Nestory Masalu, Esther Kawira, Isaac Otim, Ismail D. Legason, Leona W. Ayers, Kishor Bhatia, James J. Goedert, Ruth M. Pfeiffer, Sam M. Mbulaiteye

**Affiliations:** 1 Division of Cancer Epidemiology and Genetics, National Cancer Institute, National Institutes of Health, Department of Health and Human Services, Infections and Immunoepidemiology Branch, 9609 Medical Center Dr, Rm. 6E-118, MSC 3330, Bethesda, MD 20892 USA; 2 EMBLEM Study, St. Mary’s Hospital, Lacor, Gulu & African Field Epidemiology Network, Kampala, Uganda; 3 EMBLEM Study, Kuluva Hospital, Arua & African Field Epidemiology Network, Kampala, Uganda; 4 Division of Intramural Research, National Institute of Allergy and Infectious Diseases, National Institutes of Health, Bethesda, MD USA; 5 EMBLEM Study, Moi University College of Health Sciences, Eldoret, Kenya; 6 Moi Teaching and Referral Hospital (MTRH), Academic Model Providing Access To Healthcare (AMPATH), Eldoret, Kenya; 7 EMBLEM Study, Bugando Medical Center, Mwanza, Tanzania; 8 EMBLEM Study, Shirati Health and Educational Foundation, Shirati, Tanzania; 9 Department of Pathology, The Ohio State University, Columbus, OH USA

**Keywords:** *Plasmodium falciparum*, Epstein-Barr virus, Burkitt lymphoma, Epidemiology

## Abstract

**Background:**

*Falciparum* and endemic Burkitt lymphoma (eBL) are co-endemic in Africa, but the malaria experience in eBL patients is unknown. A lower prevalence of *falciparum* has been reported in eBL patients, but those results are anecdotally attributed to pre-enrollment anti-malaria treatment.

**Methods:**

We studied 677 eBL patients and 2920 community controls aged 0–15 years enrolled in six regions in Uganda, Tanzania, and Kenya during 2010–2016. *Falciparum* was diagnosed using thick blood film microscopy (TFM) and antigen-capture rapid diagnostic tests (RDTs). Guardians of the children answered a 40-item structured questionnaire about their child’s pre-enrollment lifetime malaria history and treatment, demographics, socioeconomics, animal exposures, fevers, and hospitalizations. We utilized exploratory factor analysis to reduce the 40 questionnaire variables into six factors, including Inpatient malaria and Outpatient malaria factors that were surrogates of pre-enrollment anti-malaria treatment. The six factors accounted for 83–90% of the variance in the questionnaire data. We calculated odds ratios and 95% confidence intervals (OR 95% CI) of association of eBL with *falciparum* positivity, defined as positive both on TFM or RDTs, or only RDTs (indicative of recent infection) or TFM (indicative of current *falciparum* infection) versus no infection, using multivariable logistic regression, controlling for group of age (0–2, 3–5, 6–8, 9–11 and 12–15 years), sex, and study site and the afore-mentioned pre-enrollment factors.

**Results:**

The prevalence of *falciparum* infection was 25.6% in the eBL cases and 45.7% in community controls (aOR = 0.43, 95% CI: 0.40, 0.47; *P* < 0.0001). The results were similar for recent *falciparum* infection (6.9% versus 13.5%, aOR = 0.44, 95% CI: 0.38, 0.50; *P* < 0.0001) and current *falciparum* infection (18.7% versus 32.1%, aOR = 0.47, 95% CI: 0.43, 0.51; *P* < 0.0001). These aORs for any, recent and current *falciparum* infection did not change when we adjusted for pre-enrollment factors (aORs = 0.46, =0.44, and = 0.51, respectively) were significantly lower in stratified analysis for any infection in children < 5 years (aOR = 0.46; 95% CI: 0.29, 0.75) or ≥ 10 years (aOR = 0.47; 95% CI: 0.32, 0.71).

**Conclusion:**

Our study results reduce support for pre-enrollment antimalaria treatment as a sole explanation for the observed lower *falciparum* prevalence in eBL cases and open a space to consider alternative immunology-based hypotheses.

**Supplementary Information:**

The online version contains supplementary material available at 10.1186/s13027-021-00377-0.

## Introduction

Endemic Burkitt lymphoma (eBL) is an aggressive germinal-center (GC) B-cell non-Hodgkin lymphoma [1] first described in African children by Denis Burkitt in 1958 [2]. Epidemiological studies noted a geographical correlation of *Plasmodium falciparum* with eBL, which opened the hypothesis about whether this correlation was evidence of causation [3]. This hypothesis has been supported by findings that eBL patients are more likely than controls to have evidence of prior malaria, as measured by higher anti-malaria antibody titers in plasma [4–6], and less likely to be protected from malaria, as measured by carriage of genetic variants that protect against malaria [7, 8]. A causal role is also supported by immunological data. Recurrent *falciparum* infection stimulates proliferation of polyclonal B cells in the GC, where BL originates, and activates the *MYC* oncogene in B cells by promoting chromosomal translocations of *MYC* on chromosome 8q24 into the vicinity of immunoglobulin genes [9, 10]. Furthermore, *falciparum* preferentially stimulates B cells infected with Epstein-Barr virus via the cysteine-rich inter-domain region 1 alpha (CIDR1alpha) of the *Plasmodium falciparum* membrane protein 1 (*Pf*EMP1) [11, 12].

However, as *falciparum* infection is associated with high malaria morbidity and mortality in children below age 3 years who lack immunity [13–15], it is unclear where eBL falls in spectrum of immunity to malaria. Malaria morbidity is due to blockage of vessels due to parasite sequestration and inflammatory response and loss of red blood cells (rbcs) [16]. Acquired immunity blocks sequestration, inflammation, and parasite infection of rbcs and promotes splenic clearance of parasites [17], although people remain susceptible and suffer frequent asymptomatic microscopic or submicroscopic infection [18]. The observation that the incidence of eBL peaks in children aged 5–9 years [19] suggests that eBL occurs in children who may have adequate protective immunity [20], but this possibility has not been fully tested [21].

Two studies reported a lower prevalence of *falciparum* in eBL patients than controls in Uganda [22] and Kenya [23]. This unexpected result was attributed to pre-enrollment anti-malaria treatment, although this hypothesis was not tested because pre-enrollment data were not collected. However, we recently observed this unexpected result (odds ratio [OR] of RDT positivity = 0.26, *p* < 0.0001) in our study of 697 eBL cases and 2934 community controls enrolled in the Epidemiology of Burkitt lymphoma in East African children and minors (EMBLEM) study in Uganda, Tanzania, and Kenya [24]. These results were not explained by malaria-related fevers reported up to 6 months pre-enrollment, which were lower in eBL cases than controls (OR = 0.47, *p* < 0.0001) or by other variables that captured pre-enrollment anti-malaria treatment. These results challenged the hypothesis that the lower prevalence of *falciparum* infection is due to pre-enrollment treatment of malaria, while also raising the possibility that *falciparum* prevalence is low in children with eBL because they have robust acquired resistance to *falciparum*.

In the current study, we conducted a more comprehensive adjustment of pre-enrollment exposures by using exploratory factor analysis (EFA) to reduce 40 questionnaire variables about the child’s pre-enrollment lifetime malaria history and treatment, demographics, socioeconomics, animal exposures, fevers, and hospitalizations into a few informative factors [25] and used those obtained factors to control for pre-enrollment anti-malaria treatment. EFA was suitable because it allowed us to adjust for all information without the risk of overfitting the data [26].

## Methods

### Population and setting

The study population has been described previously [24]. Briefly, eBL cases aged 0–15 years and community controls of a similar age, sex, and geographical area were enrolled in six regions in Uganda, Tanzania, and Kenya [24]. Both eBL cases and controls were tested for *falciparum* infection using thick blood film microscopy (TFM) and commercial antigen rapid diagnostic tests (RDTs [24]. Pre-enrollment malaria history and treatment, demographics, socioeconomics, animal exposures, fevers, and hospitalizations was elicited by questionnaire (https://emblem.cancer.gov/resources/EMBLEM_Interview_Questionnaire.pdf) (Supplementary Table 1).

### Statistical methods -- imputation, exploratory factor analysis, association analyses

The 40 data elements analyzed in this study are shown in Supplementary Table 1. Because complete data are required for EFA, missing data were imputed using sequential regression imputation with 5 imputed datasets created in IVEware (http://www.isr.umich.edu/src/smp/ive) in SAS 9.4 [27]. Imputation affected < 1% of subjects and < 10% of individual data elements where data were missed/not collected. The imputations were performed for controls and cases separately using combined and country specific datasets. The imputation coefficients were combined using Rubin’s rules in Proc MIANALYZE in SAS [28].

**Fig. 1:**
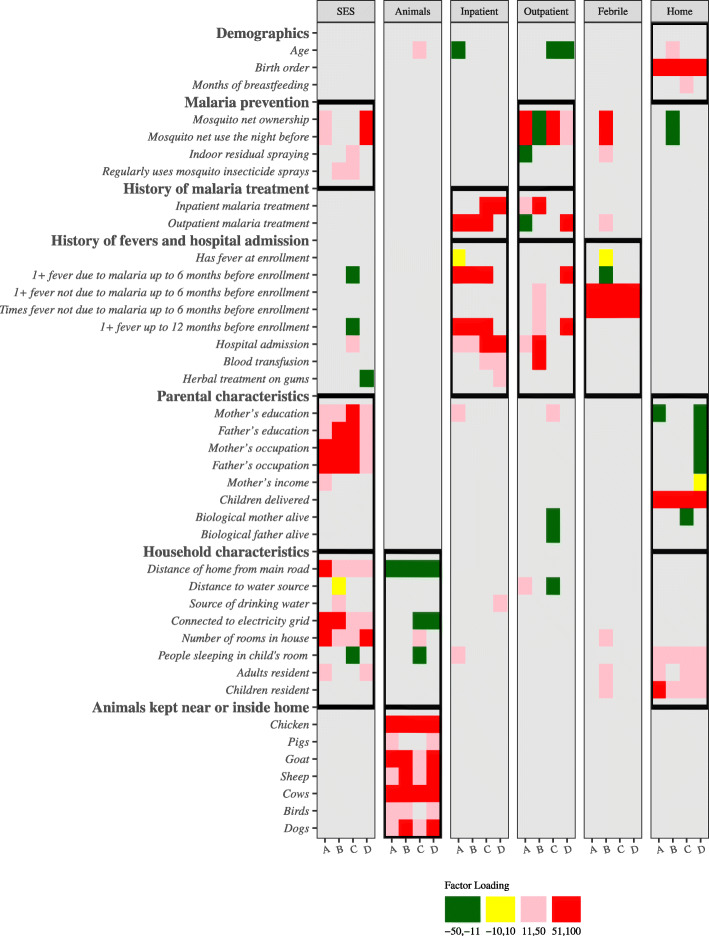
Heatmap showing factors obtained from exploratory factor analysis (EFA). The rows are questionnaire variables and the columns are the factors. The questionnaire variables are grouped into 7 domains, and the columns are the six factors that were extracted. The factors are presented country-specifically A-C in the following order: Uganda, Tanzania, and Kenya, and D for all countries combined (primary analysis). For each factor, the results are based on five imputed datasets (see Supplementary Table 2). For simplicity, the factor loadings are color coded, with significant factor loadings that have a positive value coded red and pink, while those with a significant negative value are coded light green, and dark green, and those with significant but comparatively small absolute factor loadings are coded yellow. Non-significant factor loadings are shaded gray. Domains contributing to each of the six factors are outlined with a thick black box. The scale bar shows the correlation between variables and the factor loadings

EFA was performed on the complete combined and country-specific datasets (Supplementary Table 2) and yielded six factors that we extracted using the principal component method and according to Kaiser’s rule (eigenvalue > 1) [29]. The six factors were transformed using the varimax rotation method to obtain orthogonal (uncorrelated) factors. To facilitate presentation and interpretation of the results, the extracted factors were named according to the questionnaire variables based on the highest absolute factor loadings contributing to the factor and colors used to improve visualization (as shown in Fig. 1). The factor with high (red, positive value) loading from number of rooms in the house and bed net (ownership and use) was named Socioeconomic status; the factor with high (red) loading from keeping animals inside or near the house (all except pigs and birds) was named the Animals factor; the factor with high (red) loading from the inpatient malaria treatment and hospital admission was named the Inpatient malaria factor; the factor with high (red) loading from outpatient malaria treatment and fever (within 6 or 12 months of study enrollment) was named the Outpatient malaria factor; the factor with high (red) loadings from fevers not due to malaria was named the Non-specific fevers factor. Non-specific fevers may be due to undiagnosed viral or parasitic infections [30] or due to fevers of undetermined origin in patients with cancer, also known as B-symptoms [31]. The factor with high (red) loading for birth order (number of children) and inverse (dark green) loading for maternal and paternal education and occupation was named the Home factor.

We calculated crude and adjusted ORs and 95% confidence intervals (CIs) to estimate the association of eBL with *falciparum* using multivariable logistic regression. We adjusted for group of age (0–2, 3–5, 6–8, 9–11 and 12–15 years), sex, and study site, which were considered *apriori* as confounders. *Falciparum* infection was defined two ways:  First, as a two-level variable with  no infection, defined as negative on both TFM and RDT (coded 0), or infected, defined as positive either on TFM or RDT (coded 1). Second, as a three-level variable with no infection defined as before; recent infection defined as positive on RDT but negative on TFM (coded 1), or current infection defined as positive on TFM regardless of RDT result (coded 2; Supplementary Table 1). These definitions allowed us to classify individuals as having any infection (current or recent; definition 1) and distinguish them from those with recent infection, eg, asmay be observed  in children who have been treated for malaria but still have degraded *falciparum* antigens in their blood for 3–4 weeks after treatment of malaria [32] and from those with current infection in whom blood-stage parasite forms are visualized. If the previously reported inverse association of *falciparum* with eBL is due to pre-enrollment anti-malaria treatment, we would expect to observe an inverse association between eBL and current *falciparum* infection but a positive association betwen eBL and recent *falciparum* infection. Additionally, we would expect that adjustment for pre-enrollment anti-malaria treatment would attenuate or eliminate the association of eBL with measures of *falciparum* infection. We report the combined results primarily and country-specific results secondarily to explore the consistency of effects across the three countries.

## Results

### Characteristics of included children

**Fig. 2:**
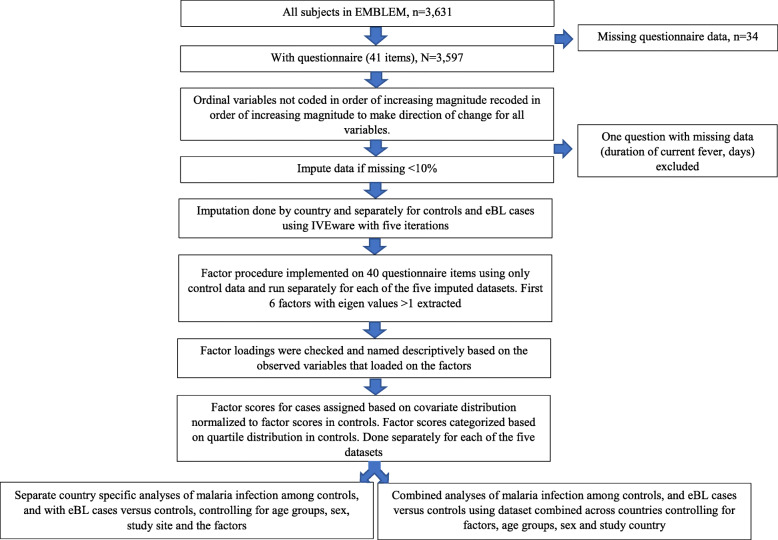
Flowchart showing the study process of selection of subjects, data checking, imputation of missing data, and case-control analysis

We analyzed 3631 children (692 eBL cases) enrolled in Uganda, Tanzania, and Kenya during 2010–2016 (Fig. 2). Of these, 34 children (15 eBL patients) were excluded because they were missing all questionnaire data. The remaining 3597 children (677 eBL cases; 62% with pathology confirmation) were  included in the EFA. Table 1 shows the characteristics of the eBL cases and community controls analyzed. Overall, 55.3% were male, 12.2% had recent *falciparum* infection and 29.6% had current *falciparum* infection. Uganda contributed most (41%), followed by Kenya (33%), and the least number was from Tanzania (26%).

**Table 1:** Characteristics of controls and eBL cases 0–15 years in the EMBLEM study

Characteristics	Controls, n (column %)	Cases, n (column %)	All, n (column %)
Country			
Uganda	1146 (39.3)	315 (46.5)	1461 (40.6)
Tanzania	816 (28.0)	127 (18.8)	943 (26.2)
Kenya	958 (32.8)	235 (34.7)	1193 (33.2)
*P*-value		**< 0.0001**	
Age, years			
0–2	193 (6.6)	65 (9.6)	258 (7.2)
3–5	698 (23.9)	177 (26.1)	875 (24.3)
6–8	913 (31.3)	188 (27.8)	1101 (30.6)
9–11	668 (22.9)	145 (21.4)	813 (22.6)
12–15	448 (15.3)	102 (15.1)	550 (15.3)
*P*-value		**0∙03**	
Male	1563 (53.5)	427 (63.1)	1990 (55.3)
*P*-value		**< 0.0001**	
Malaria results^a^			
Negative	1569 (54.4)	480 (74.4)	2049 (58.0)
Positive (Antigens or parasites)	1318 (45.7)	165 (25.6)	1483 (42.0)
Missing	33	32	65
*P*-value		**< 0.0001**	
Malaria result^a^			
Negative	1564 (54.5)	478 (74.5)	2042 (58.2)
Recent (Only RDT positive)	384 (13.4)	44 (6.9)	428 (12.2)
Current (TFM positive)	920 (32.1)	120 (18.7)	1040 (29.6)
Missing	52	35	87
*P*-value		**< 0.0001**	
Questionnaire Data			
Available	2920 (99.5)	677 (97.1)	3597 (99.1)
Missing	14 (0.5)	20 (2.9)	34 (0.9)
*P*-value		**< 0.0001**	

Children with missing questionnaire data were not included in the computation of the frequencies presented in the table

^a^Percentages for malaria results,  Microscopy- and RDT-defined infection, do not add to 100% because some subjects are missing one set of results

### Association of the factors with *falciparum* among community controls

Table 2 shows, for all study sites combined, the univariate and adjusted associations with *falciparum* infection with the factors among the controls. Higher scores on the Socioeconomic status and Inpatient malaria factors were inversely associated with *falciparum* positivity. The association of *falciparum *infection with the Socioeconomic factor suggested a monotonic trend (aOR was 0.79 for Q2 vs. Q1, declining to aOR 0.45 for Q4 vs. Q1; *P* < 0.0001), whereas the association of *falciparum* infection with the Inpatient malaria factor was more consistent with a threshold (vs Q1 aORs were 0.75 for Q2, 0.79 for Q3, and 0.71 for Q4). Higher scores on the Animals, Non-specific fevers, and Home factors were associated with *falciparum* positivity, with a pattern suggesting monotonic trend for the Animals factor (aORs was 1.28 for Q2 rising to 1.69 for Q4 vs. Q1; *P* < 0.0001) and a threshold for the Non-specific fevers (aORs were 1.29 to 1.40 for Q2 to Q4 vs. Q1) and the Home factors (aORs were 1.14 to 1.38 for Q2 to Q4 vs. Q1). The Outpatient factor was unrelated to *falciparum* positivity (*P* = 0.20). Although there were slight variations, the associations observed in the combined analyses were present in country-specific analyses (Supplementary Table 3).

**Table 2:** Odds ratios and 95% confidence intervals (CIs) of the association of factors with falciparum infection risk in controls and endemic Burkitt lymphoma in the cases and controls in the combined dataset

Factor	Falciparum infection		Endemic Burkitt lymphoma	
	OR (95% CI)^**a**^	aOR (95% CI) ^**b**^	OR (95% CI)^**a**^	aOR (95% CI) ^**b**^
Socioeconomic status *Q1*	Ref	Ref	Ref	Ref
*Q2*	0.79 (0.63, 0.99)	0.79 (0.62, 1.01)	1.68 (1.27, 2.22)	2.32 (1.68, 3.20)
*Q3*	0.65 (0.52, 0.81)	0.62 (0.48, 0.80)	1.63 (1.24, 2.14)	2.25 (1.58, 3.21)
*Q4*	0.44 (0.36, 0.55)	0.45 (0.34, 0.59)	2.13 (1.64, 2.77)	3.25 (2.28, 4.65)
*P-trend*	**< 0.0001**	**< 0.0001**	**< 0.0001**	**< 0.0001**
Animals *Q1*	Ref	Ref	Ref	Ref
*Q2*	1.25 (1.02, 1.54)	1.28 (1.03, 1.59)	1.20 (0.94, 1.53)	1.23 (0.93, 1.62)
*Q3*	1.37 (1.10, 1.69)	1.41 (1.12, 1.76)	1.02 (0.79, 1.31)	1.14 (0.85, 1.52)
*Q4*	1.52 (1.23, 1.87)	1.69 (1.36, 2.11)	1.44 (1.14, 1.82)	1.72 (1.31, 2.25)
*P-trend*	**< 0.0001**	**< 0.0001**	**0.01**	**0.0002**
Inpatient malaria *Q1*	Ref	Ref	Ref	Ref
*Q2*	0.77 (0.63, 0.95)	0.75 (0.60, 0.94)	1.94 (1.44, 2.60)	2.09 (1.50, 2.89)
*Q3*	0.83 (0.67, 1.02)	0.79 (0.62, 1.01)	2.73 (2.03, 3.68)	2.69 (1.88, 3.84)
*Q4*	0.78 (0.63, 0.97)	0.71 (0.56, 0.91)	2.99 (2.24, 3.98)	2.14 (1.51, 3.04)
*P-trend*	0.05	**0.007**	**< 0.0001**	**< 0.0001**
Outpatient malaria *Q1*	Ref	Ref	Ref	Ref
*Q2*	1.01 (0.82, 1.25)	0.90 (0.72, 1.12)	1.40 (1.08, 1.81)	1.24 (0.92, 1.66)
*Q3*	1.17 (0.95, 1.45)	1.05 (0.84, 1.31)	1.16 (0.89, 1.51)	1.16 (0.86, 1.56)
*Q4*	1.01 (0.82, 1.24)	1.08 (0.85, 1.36)	1.82 (1.43, 2.32)	1.75 (1.31, 2.33)
*P-trend*	0.60	0.20	**< 0.0001**	**0.0004**
Non-specific febrile symptoms *Q1*	Ref	Ref	Ref	Ref
*Q2*	1.44 (1.16, 1.79)	1.35 (1.08, 1.71)	0.81 (0.60, 1.09)	1.26 (0.91, 1.75)
*Q3*	1.35 (1.09, 1.67)	1.29 (1.01, 1.64)	0.75 (0.55, 1.03)	1.53 (1.08, 2.17)
*Q4*	1.27 (1.03, 1.58)	1.40 (1.09, 1.78)	3.49 (2.75, 4.43)	7.30 (5.45, 9.79)
*P-trend*	0.06	**0.009**	**< 0.0001**	**< 0.0001**
Home environment *Q1*	Ref	Ref	Ref	Ref
*Q2*	1.29 (1.05, 1.59)	1.20 (0.97, 1.50)	0.96 (0.74, 1.23)	1.27 (0.95, 1.70)
*Q3*	1.22 (0.98, 1.52)	1.14 (0.91, 1.44)	1.19 (0.94, 1.52)	1.61 (1.21, 2.13)
*Q4*	1.38 (1.12, 1.70)	1.38 (1.10, 1.73)	1.29 (1.01, 1.63)	1.83 (1.38, 2.44)
*P-trend*	**0.005**	**0.008**	**0.01**	**0.0003**
Malaria infection				
TFM or RDT results	**–**	**–**		
Negative on both tests	**–**	**–**	Ref	Ref
TFM or RDT positive	**–**	**–**	0.43 (0.40, 0.47)	0.46 (0.37, 0.56)
Malaria infection				
Negative on both tests	**–**	**–**	Ref	Ref
Recent (only RDT positive)	**–**	**–**	0.44 (0.38, 0.50)	0.44 (0.29, 0.65)
Current (TFM positive)	**–**	**–**	0.47 (0.43, 0.51)	0.51 (0.39, 0.66)
*P-trend*			**< 0.0001**	**< 0.0001**

^a^Crude odds ratios, ^b^Model adjusted further for age using five groups (0–2, 3–5, 6–8, 9–11 and 12–15 years with dummy variables), sex and study region and the six extracted factors (SES, Animals, Inpatient malaria, Outpatient malaria, Non-specific fevers and Home factor); *TFM* Thick film microscopy, *RDT* Rapid diagnostic test

### Association of the factors with eBL

**Fig. 3:**
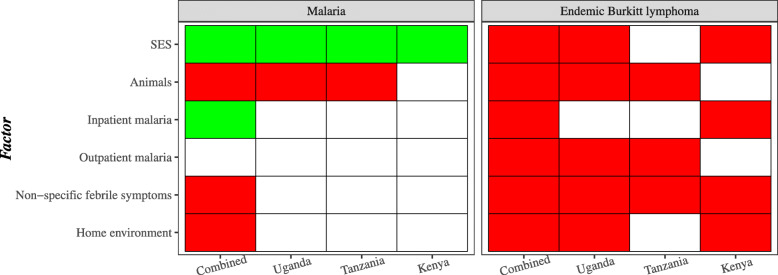
Heat map showing direction of associations with *P* < 0.05 between extracted factors and malaria infection in the controls and with eBL in the case-control analysis for both the combined and country-specific analysis, which are separated by dark column bars. The green color indicates an inverse association (lower risk), red color indicates a positive association (increased risk), while no coloring indicates a null association. For example, the SES factor was inversely associated with *falciparum* infection, but positively associated with eBL risk

All the factors were associated with increased odds of eBL in crude and adjusted analyses that included the factors (Table 2). Although *falciparum* positivity was inversely related to the Socioeconomic status and Inpatient malaria factors, these two facors were associated with higher odds of eBL (Table 2, Fig. 3). However, the associations of eBL with the Animals, Non-specific fevers, and Home factors were consistent, ie, in the same direction as the associations of those factors with *falciparum* positivity, while the Outpatient malaria factor, which was unrelated to *falciparum* positivity, was associated with higher odds of eBL (Fig. 3).

### Association of *falciparum* positivity with eBL diagnosis

We observed lower *falciparum* positivity in eBL cases than community controls (25.6% versus 45.7%, *P* < 0.0001) resulting in lower odds of eBL (aOR = 0.43, 95% CI: 0.40, 0.47, Table 2) in those with infection, in analyses adjusting for group of age, sex, and study site. The associations did not change when we included the aforementioned factors in the logistic regression model to control for pre-enrollment anti-malaria treatment (aOR = 0.46, 95% CI: 0.37, 0.56). The inverse association remained robust (aOR = 0.45, 95% CI 0.35, 0.59, *P* < 0.0001) when we further adjusted for mean platelet count, hemoglobin, and WBC count as confounders [33] and in stratified analysis for children < 5 years (aOR = 0.46; 95% CI: 0.29, 0.75) and those ≥10 years (aOR = 0.47; 95% CI: 0.32, 0.71), and was significant in country-specific analyses (aOR = 0.54 in Uganda, aOR = 0.48 in Tanzania and aOR = 0.30 in Kenya; Supplementary Table 4).

The adjusted results were similar for recent *falciparum* infection (aOR = 0.44, 95% CI 0.29, 0.65, *P* < 0.0001) or current *falciparum* infection (aOR = 0.51, 95% CI 0.39, 0.66, *P* < 0.0001).

## Discussion

Our study found significantly lower prevalence of *falciparum* positivity, defined as any, recent, or current *falciparum* infection, in eBL patients than controls in crude and adjusted analyses. The ORs did not change when we adjusted for Inpatient malaria and Outpatient malaria factors as surrogates of pre-enrollment anti-malaria treatment, for the other four factors obtained from EFA, for platelets, low hemoglobin, and elevated white cell counts, which are risk factors for eBL [33]. The results were significant in stratified analyses by age (< 5 years versus ≥10 years or older), which is a surrogate for age-related acquired immunity against malaria. These results are similar to those reported in two studies that did not control for pre-enrollment anti-malaria treatment [23, 34] and those in our previous analyses that included a limited adjustment for a few variables about pre-enrollment anti-malaria treatment [24, 33]. Our findings, based on the most comprehensive adjustment of pre-enrollment exposures and for other risk factors of eBL and stratified analyses, reduce support for the null hypothesis that pre-enrollment anti-malaria treatment is the major explanation for the observed low *falciparum* positivity among eBL patients, on one hand,  and increase support for exploring alternative hypotheses, on the other hand.

One alternative hypothesis, that is usually dismissed  because it is counter-intuitive, is that lower prevalence of *falciparum* infection in eBL children is a surrogate of robust immunity to malaria. Immune response to *falciparum* spans a spectrum from lack of immunity, which is associated with severe malaria to strong age-related acquired anti-malaria and anti-parasite immunity [13], which is associated with mild or asymptomatic *falciparum* infection. We suggest from our results that eBL cases possess strong anti-malaria and anti-parasite immunity. Lack of immunity represents a serious risk of death from severe malaria in areas with high eBL incidence, thus, immunity against severe malaria usually develops quickly, in some estimates occurs after exposure to 1–2 *falciparum* infections and is usually well-developed in the first 1–3 years of life in malaria endemic countries [13]. However, immunity against severe malaria does not prevent *falciparum* infection or the development of mild/moderate malaria, which are also associated with serious life-threatening morbidity and mortality from anemia, stunting, malnutrition, bacterial co-infections [35], and seizures [36]. The risk for these complications reduces progressively with  acquisition of age-related anti-malaria and anti-parasite immunity at a rate of 6% per year of age and 2% per episode of clinical malaria in some areas [14]. Despite having well-developed  anti-malaria and anti-parasite immunity by age 5 years in high malaria burden areas, low-grade mild, asymptomatic microscopic or submicroscopic infections continue to afflict children [20]. Because eBL, which by definition occurs in children afflicted by heavier malaria at a median age of 5–9 years, we infer  from this pattern that children predisposed to eBL have strong anti-parasite immunity before and after disease onset. This inference is intuitively consistent with the  prediction that children with eBL would be expected to have capacity to control *falciparum* infection to survive in those areas, and it is supported by our finding that eBL cases have significantly lower parasite density than controls of a similar age [20].

The inferences above also suggest that there are some important differences between  malaria versus viral carcinogenesis [37, 38]. In the latter, inability to control viral infection promotes inflammation and malignancy [39] and viral infections can be grouped into high-risk versus low-risk types depending on their association with cancer risk, e.g. cervical cancer and high-risk human papillomavirus types (eg, 16 or 18) [40]. This model does not apply to *falciparum* because lack of immunity to malaria would predispose to severe malaria and high risk of death before eBL [41]. Thus, children who develop immunity to severe malaria relatively quickly and  acquire immunity to resist new infections [13] as a central strategy to thrive in hostile environments with heavy exposure to *falciparum* parasites [42]. Our studies in Ghana and Uganda have confirmed that eBL children as well as similarly aged controls have high antibody titers against *falciparum* variants linked to severe malaria, indicating prior exposure to and immune response to such variants [6]. Although the development of immunity is hierarchical, is correlated with age-dependent antibodies, and progressively limits parasitemia [43], it does not prevent infection and development of low-grade asymptomatic or sub-microscopic *falciparum *parasitemia [44]. Children with immunity to malaria have a lower risk of death from severe malaria [16, 18], but they are exposed to  chronic *falciparum* parasitemia  and increased activity by splenic macrophages to clear parasites [17]. We suggest that this increased *falciparum* antigenic load to the spleen is a risk factor for eBL [45] in children in malaria endemic regions. We further infer that it is not high-risk *falciparum* variants (as is the case for severe malaria), but  the multiplicity of diverse variants the children are exposed to that are the risk factor for eBL [46]. *Falciparum* parasites can establish  new infections in immune populations only by allelic switching of *falciparum* multigene families, such as *Pf*EMP1 [47], to express new epitopes that are new to a particular host and, therefore, not recognized by the immunity acquired by that host up to that time. Our recent studies are consistent with this notion that infection with multiple (swarms of) *falciparum* variants, presumably expressing novel epitopes that are not recognized by the hosts' acquired immunity, may be related to eBL [19, 48, 49].

Our results highlight potential value of establishing collaboration between malaria and eBL research to jointly address  overlapping interests. A  better appreciation of the link between eBL and exposure to high *falciparum* burden could encourage better coordination between eBL and malariologists. eBL may be a suitable sentinel condition to detect  pockets of high localized community *falciparum* transmission, which can be identified and targeted for investigation to suppress  reservoirs that may threaten malaria prevention and intervention programs [20]. In view of our findings that eBL is associated with multiplicity of *falciparum* infection [19, 48, 49] and recent studies suggesting that markers of *falciparum* genotypic complexity are better measures of force of falciparum infection, co-transmission and superinfection events [50], close collaboration could facilitate the discovery of reliable markers of *falciparum* complexity that could also be used as an intermediate phenotype for eBL.

The strengths of our study include having a large sample size, data from three countries, detailed data about anti-malaria treatment before study enrollment, and measurement of *falciparum* using two methods. Our use of EFA is novel in eBL-malaria research, which made it possible for us to rigorously control for confounding without overfitting the data. The use of two tests for *falciparum* allowed us to test associations of eBL with recent anti-malaria treatment, which would have detected a positive association of recent *falciparum* with eBL, presumably because such  children may have unreported  pre-enrollment anti-malaria treatment. We acknowledge that the case-control design is a weakness of our study because it is susceptible to reverse causality that cannot be fully addressed. Nonetheless, our results substantially increase our understanding about the relationship between eBL and *falciparum* infection, the role of pre-enrollment exposures, and offer support for considering novel hypotheses.

In conclusion, we show that *falciparum* infection was significantly lower in eBL cases than controls in crude and adjusted analyses. The results were robust in analyses controlling for pre-enrollment factors and in analyses stratified by age. These results reduce support for pre-enrollment antimalaria treatment as a sole explanation for the observed lower *falciparum* prevalence in eBL cases, and open up space to consider alternative hypotheses such as the state of  immunity to *falciparum* infection in children before and after eBL onset.

## Supplementary Information

**Additional file 1: Supplementary Table 1.** Questionnaire variables/data elements and how they were coded for exploratory factor analysis. **Supplementary Table 2.** Eigenvalue and variance explained by extracted factors shown for each imputed dataset and by study country. **Supplementary Table 3.** Odds ratios and 95% confidence intervals (CIs) of the association of factors with falciparum infection risk in controls, by country. **Supplementary Table 4.** Odds ratios and 95% confidence intervals (CIs) of the association of factors with eBL risk, by country.

## Data Availability

Data and code used in the current analysis will be made available upon request from the corresponding author.

## Abbreviations

CIDR1alpha: Cysteine-rich inter-domain region 1 alpha
EMBLEM: Epidemiology of Burkitt lymphoma in East African children and minors
EFA: Exploratory factor analysis
eBL: endemic Burkitt lymphoma
OR: Odds ratio
95% CI: 95% confidence interval
RDTs: Rapid diagnostic tests
*Pf*EMP1: *Plasmodium falciparum* membrane protein 1
Q: Quartile
TFM: Thick film microscopy
