# Supplementary material for: Inverse association of falciparum positivity with endemic Burkitt lymphoma is robust in analyses adjusting for pre-enrollment malaria in the EMBLEM case-control study
Source: Infect Agent Cancer. 2021 Jun 7;16:40. doi: 10.1186/s13027-021-00377-0 (PMC8186042; doi:10.1186/s13027-021-00377-0)
Supplement: Supplementary file 1 — Additional file 1: Supplementary Table 1. Questionnaire variables/data elements and how they were coded for exploratory factor analysis. Supplementary Table 2. Eigenvalue and variance explained by extracted factors shown for each imputed dataset and by study country. Supplementary Table 3. Odds ratios and 95% confidence intervals (CIs) of the association of factors with falciparum infection risk in controls, by country. Supplementary Table 4. Odds ratios and 95% confidence intervals (CIs) of the association of factors with eBL risk, by country. [file 13027_2021_377_MOESM1_ESM.docx]

**Supplementary Table 1.** Questionnaire variables/data elements and how they were coded for exploratory factor analysis

| **Domain** | **Variable name** | **Range** | **Coding** |
| --- | --- | --- | --- |
| Demographics | Age, years | 0-15 years | Continuous in single years |
|  | Sex |  | 0=Female and 1=Male |
|  | Birth order | 1-6 | 1, 2, 3, 4, 5 and 6+ |
|  | Months of breast feeding | 0-84 | 0-12, 13-24 and 25+ |
| Malaria prevention | Mosquito net ownership |  | 1=No and 2=Yes |
|  | Mosquito net use the night before |  | 1=No and 2=Yes |
|  | Indoor residual insecticide sprayed in house |  | 1=No and 2=Yes |
|  | Regularly uses mosquito insecticide sprays |  | 1=No and 2=Yes |
| History of malaria treatment | Inpatient malaria treatment |  | 1=Never, 2=Yes, past 12 months, 3=Yes, 13-24 months and 4= Yes, > 24 months |
|  | Outpatient malaria treatment * |  | 1=Never and 2=Yes |
| History of fevers and hospital admission | Has fever at enrollment |  | 1=No and 2=Yes |
|  | >1 fever due to malaria in the past 6 months |  | 1=No and 2=Yes |
|  | >1 fever not due to malaria in the last 6 months |  | 1=No and 2=Yes |
|  | Times fever not due to malaria in the last 6 months | 0-2 | 0, 1-2 and 3+ |
|  | ≥1 fever in the last 12 months |  | 1=No and 2=Yes |
|  | >1 hospital admission | 0-3 | 0, 1, 2, 3 and 4+ |
|  | Ever received a blood transfusion |  | 1=No and 2=Yes |
|  | Received herbal treatment on gums |  | 1=No and 2=Yes |
| Parental characteristics | Mother’s education |  | 1=None, 2=Up to standard 4, 3=Standard 5-7 and 4=≥ Senior secondary school |
|  | Father’s education |  | 1=None, 2=Up to standard 4, 3=Standard 5-7 and 4=≥ Senior secondary school |
|  | Mother’s occupation |  | 1=Farmer, 2=Trader/sales, 3=Professional/clerical and 4=Household/other |
|  | Father’s occupation |  | 1=Farmer, 2=Trader/sales, 3=Professional/clerical and 4=Household/other |
|  | Standardized mother’s income§ |  | Standardized to mean mother’s income in each country |
|  | Number children ever delivered |  | 1, 2, 3, 4, 5 and 6+ |
|  | Biological mother alive |  | 1=Alive and 2=Deceased |
|  | Biological father alive |  | 1=Alive and 2=Deceased |
| Home characteristics | Distance of home from main road |  | 1= Far from the main road, 2= Near the main road and 3=Town/City |
|  | Distance to water source, meters |  | 1=< 500, 2=500-999, 3=1000-4999 and 4=≥ 5000 |
|  | Source of drinking water |  | 1=Unprotected spring/well, 2=Protected spring/well, 3=Public tap/piped home and 4=Other |
|  | Connected to electricity grid |  | 1=No and 2=Yes |
|  | Number of rooms in house | 1-3 | 1, 2 and 3+ |
|  | Number of people sleeping in the same room as child | 1-4 | 0-2, 3, 4 and 5+ |
|  | Number of adults resident | 1-3 | 1, 2 and 3+ |
|  | Number of children resident | 1-3 | 1, 2 and 3+ |
| Animals kept near or inside house | Chicken |  | 1=No and 2=Yes |
|  | Pigs |  | 1=No and 2=Yes |
|  | Goat |  | 1=No and 2=Yes |
|  | Sheep |  | 1=No and 2=Yes |
|  | Cows |  | 1=No and 2=Yes |
|  | Birds |  | 1=No and 2=Yes |
|  | Dogs |  | 1=No and 2=Yes |

*Due to small numbers the “Outpatient malaria treatment” variable was categorized as a binary yes/no variable

**Supplementary Table 2.** Eigenvalue and variance explained by extracted factors shown for each imputed dataset and by study country.

| **Factor** | **Imputation dataset 1** | | | **Imputation dataset 2** | | | **Imputation dataset 3** | | | **Imputation dataset 4** | | | **Imputation dataset 5** | | |
| --- | --- | --- | --- | --- | --- | --- | --- | --- | --- | --- | --- | --- | --- | --- | --- |
|  | **Eigenvalue** | **Proportion** | **Cumulative** | **Eigenvalue** | **Proportion** | **Cumulative** | **Eigenvalue** | **Proportion** | **Cumulative** | **Eigenvalue** | **Proportion** | **Cumulative** | **Eigenvalue** | **Proportion** | **Cumulative** |
| **Combined** |  |  |  |  |  |  |  |  |  |  |  |  |  |  |  |
| SES | 3∙19 | 0∙27 | 0.27 | 3∙19 | 0∙27 | 0.27 | 3∙19 | 0∙27 | 0.27 | 3∙19 | 0∙27 | 0.27 | 3∙19 | 0∙27 | 0.27 |
| Animals | 1∙95 | 0∙16 | 0.43 | 1∙96 | 0∙17 | 0.44 | 1∙96 | 0∙16 | 0.43 | 1∙96 | 0∙16 | 0.43 | 1∙98 | 0∙17 | 0.44 |
| Inpatient malaria | 1∙28 | 0∙11 | 0.54 | 1∙28 | 0∙11 | 0.55 | 1∙28 | 0∙11 | 0.54 | 1∙28 | 0∙11 | 0.54 | 1∙28 | 0∙11 | 0.55 |
| Outpatient malaria | 1∙12 | 0∙09 | 0.63 | 1∙12 | 0∙09 | 0.64 | 1∙12 | 0∙09 | 0.63 | 1∙11 | 0∙09 | 0.63 | 1∙12 | 0∙09 | 0.64 |
| Non-specific febrile symptoms | 1∙40 | 0∙12 | 0.75 | 1∙41 | 0∙12 | 0.76 | 1∙41 | 0∙12 | 0.75 | 1∙40 | 0∙12 | 0.75 | 1∙40 | 0∙12 | 0.76 |
| Home environment | 1∙64 | 0∙14 | 0.89 | 1∙65 | 0∙14 | 0.90 | 1∙64 | 0∙14 | 0.89 | 1∙64 | 0∙14 | 0.89 | 1∙64 | 0∙14 | 0.90 |
| **Uganda** |  |  |  |  |  |  |  |  |  |  |  |  |  |  |  |
| SES | 3∙36 | 0∙25 | 0∙25 | 3∙37 | 0∙24 | 0∙24 | 3∙40 | 0∙25 | 0∙25 | 3∙39 | 0∙25 | 0∙25 | 3∙37 | 0∙24 | 0∙24 |
| Animals | 1∙89 | 0∙14 | 0∙38 | 1∙91 | 0∙14 | 0∙38 | 1∙91 | 0∙14 | 0∙38 | 1∙93 | 0∙14 | 0∙38 | 1∙90 | 0∙14 | 0∙38 |
| Inpatient malaria | 1∙73 | 0∙13 | 0∙51 | 1∙73 | 0∙13 | 0∙51 | 1∙72 | 0∙12 | 0∙51 | 1∙73 | 0∙13 | 0∙51 | 1∙72 | 0∙12 | 0∙51 |
| Outpatient malaria | 1∙61 | 0∙12 | 0∙63 | 1∙62 | 0∙12 | 0∙63 | 1∙62 | 0∙12 | 0∙63 | 1∙62 | 0∙12 | 0∙63 | 1∙62 | 0∙12 | 0∙63 |
| Non-specific febrile symptoms | 1∙46 | 0∙11 | 0∙73 | 1∙46 | 0∙11 | 0∙73 | 1∙46 | 0∙11 | 0∙73 | 1∙45 | 0∙11 | 0∙73 | 1∙46 | 0∙11 | 0∙73 |
| Home environment | 1∙30 | 0∙09 | 0∙83 | 1∙30 | 0∙09 | 0∙83 | 1∙30 | 0∙09 | 0∙83 | 1∙30 | 0∙09 | 0∙83 | 1∙30 | 0∙09 | 0∙83 |
| **Tanzania** |  |  |  |  |  |  |  |  |  |  |  |  |  |  |  |
| SES | 1∙61 | 0∙14 | 0∙14 | 1∙61 | 0∙14 | 0∙14 | 1∙61 | 0∙14 | 0∙14 | 1∙61 | 0∙14 | 0∙14 | 1∙61 | 0∙14 | 0∙14 |
| Animals | 2∙28 | 0∙19 | 0.33 | 2∙29 | 0∙19 | 0.33 | 2∙27 | 0∙19 | 0.33 | 2∙29 | 0∙19 | 0.33 | 2∙28 | 0∙19 | 0.33 |
| Inpatient malaria | 1∙08 | 0∙09 | 0.42 | 1∙09 | 0∙09 | 0.42 | 1∙09 | 0∙09 | 0.42 | 1∙09 | 0∙09 | 0.42 | 1∙08 | 0∙09 | 0.42 |
| Outpatient malaria | 1∙48 | 0∙13 | 0.55 | 1∙49 | 0∙13 | 0.55 | 1∙48 | 0∙13 | 0.55 | 1∙49 | 0∙13 | 0.55 | 1∙48 | 0∙13 | 0.55 |
| Non-specific febrile symptoms | 2∙76 | 0∙23 | 0.78 | 2∙76 | 0∙23 | 0.78 | 2∙75 | 0∙23 | 0.78 | 2∙77 | 0∙23 | 0.78 | 2∙76 | 0∙23 | 0.78 |
| Home environment | 1∙20 | 0∙10 | 0.88 | 1∙20 | 0∙10 | 0.88 | 1∙20 | 0∙10 | 0.88 | 1∙20 | 0∙10 | 0.88 | 1∙20 | 0∙10 | 0.88 |
| **Kenya** |  |  |  |  |  |  |  |  |  |  |  |  |  |  |  |
| SES | 2∙52 | 0∙20 | 0∙20 | 2∙51 | 0∙20 | 0∙20 | 2∙52 | 0∙20 | 0∙20 | 2∙51 | 0∙20 | 0∙20 | 2∙50 | 0∙20 | 0∙20 |
| Animals | 1∙86 | 0∙15 | 0.35 | 1∙86 | 0∙15 | 0.35 | 1∙87 | 0∙15 | 0.35 | 1∙87 | 0∙15 | 0.35 | 1∙87 | 0∙15 | 0.35 |
| Inpatient malaria | 1∙45 | 0∙12 | 0.47 | 1∙47 | 0∙12 | 0.47 | 1∙47 | 0∙12 | 0.47 | 1∙46 | 0∙12 | 0.47 | 1∙47 | 0∙12 | 0.47 |
| Outpatient malaria | 1∙33 | 0∙11 | 0.58 | 1∙32 | 0∙11 | 0.58 | 1∙32 | 0∙11 | 0.58 | 1∙32 | 0∙11 | 0.58 | 1∙32 | 0∙11 | 0.58 |
| Non-specific febrile symptoms | 1∙21 | 0∙10 | 0.68 | 1∙20 | 0∙10 | 0.68 | 1∙20 | 0∙10 | 0.68 | 1∙21 | 0∙10 | 0.68 | 1∙21 | 0∙10 | 0.68 |
| Home environment | 2∙18 | 0∙18 | 0.86 | 2∙19 | 0∙18 | 0.86 | 2∙19 | 0∙18 | 0.86 | 2∙19 | 0∙18 | 0.86 | 2∙20 | 0∙18 | 0.86 |

**Supplementary Table 3.** Odds ratios and 95% confidence intervals (CIs) of the association of factors with falciparum infection risk in controls, by country

| **Factor** | **Uganda** | | **Tanzania** | | **Kenya** | |
| --- | --- | --- | --- | --- | --- | --- |
|  | **OR (95% CI)*** | **aOR (95% CI)** **†** | **OR (95% CI)*** | **aOR (95% CI)** **†** | **OR (95% CI)*** | **aOR (95% CI)** **†** |
| Socioeconomic status *Q1* | Ref | Ref | Ref | Ref | Ref | Ref |
| *Q2* | 0.88 (0.63, 1.24) | 0.79 (0.55, 1.15) | 0.67 (0.45, 1.01) | 0.62 (0.41, 0.95) | 0.77 (0.53, 1.13) | 0.75 (0.50, 1.13) |
| *Q3* | 0.75 (0.53, 1.06) | 0.62 (0.42, 0.92) | 0.47 (0.31, 0.70) | 0.41 (0.27, 0.65) | 1.00 (0.69, 1.45) | 0.96 (0.64, 1.43) |
| *Q4* | 0.44 (0.31, 0.62) | 0.36 (0.25, 0.53) | 0.20 (0.13, 0.31) | 0.19 (0.12, 0.31) | 0.59 (0.40, 0.85) | 0.55 (0.36, 0.84) |
| *P-heterogeneity* | **0.0002** | **0∙0002** | **<0.0001** | **0∙00001** | **0.04** | **0∙04** |
| *P-trend* | **<0.0001** | **<0.0001** | **<0.0001** | **<0.0001** | **0.03** | **0.007** |
|  |  |  |  |  |  |  |
| Animals *Q1* | Ref | Ref | Ref | Ref | Ref | Ref |
| *Q2* | 1.37 (0.98, 1.91) | 1.32 (0.92, 1.88) | 1.23 (0.81, 1.87) | 1.08 (0.69, 1.72) | 1.25 (0.87, 1.81) | 1.27 (0.87, 1.86) |
| *Q3* | 1.20 (0.86, 1.67) | 1.22 (0.86, 1.74) | 1.57 (1.04, 2.37) | 1.61 (1.02, 2.56) | 1.05 (0.72, 1.53) | 1.15 (0.77, 1.71) |
| *Q4* | 2.31 (1.65, 3.24) | 2.65 (1.84, 3.81) | 1.66 (1.10, 2.51) | 1.97 (1.22, 3.18) | 1.22 (0.84, 1.75) | 1.31 (0.87, 1.96) |
| *P-heterogeneity* | **0.0003** | **<0∙00001** | 0.29 | **0∙03** | 0.61 | 0∙79 |
| *P-trend* | **<0.0001** | **<0.0001** | **0.008** | **0.0009** | 0.49 | 0.27 |
|  |  |  |  |  |  |  |
| Inpatient malaria *Q1* | Ref | Ref | Ref | Ref | Ref | Ref |
| *Q2* | 1.06 (0.76, 1.48) | 0.97 (0.68, 1.40) | 1.28 (0.86, 1.90) | 1.22 (0.78, 1.89) | 1.06 (0.73, 1.55) | 1.18 (0.77, 1.82) |
| *Q3* | 1.11 (0.80, 1.55) | 1.02 (0.71, 1.46) | 0.99 (0.66, 1.47) | 1.09 (0.69, 1.70) | 1.07 (0.73, 1.55) | 1.29 (0.81, 2.06) |
| *Q4* | 1.12 (0.80, 1.55) | 1.25 (0.86, 1.83) | 0.88 (0.59, 1.31) | 0.93 (0.59, 1.46) | 0.95 (0.65, 1.38) | 1.10 (0.70, 1.73) |
| *P-heterogeneity* | 0.95 | 0∙38 | 0.18 | 0∙56 | 0.78 | 0∙76 |
| *P-trend* | 0.48 | 0.27 | 0.32 | 0.96 | 0.79 | 0.83 |
|  |  |  |  |  |  |  |
| Outpatient malaria *Q1* | Ref | Ref | Ref | Ref | Ref | Ref |
| *Q2* | 2.05 (1.45, 2.90) | 1.80 (1.25, 2.60) | 0.76 (0.51, 1.15) | 0.73 (0.45, 1.19) | 0.79 (0.54, 1.16) | 0.83 (0.56, 1.24) |
| *Q3* | 1.53 (1.09, 2.14) | 1.25 (0.86, 1.83) | 1.06 (0.71, 1.57) | 1.12 (0.68, 1.84) | 0.72 (0.50, 1.03) | 0.74 (0.50, 1.10) |
| *Q4* | 1.69 (1.21, 2.37) | 1.24 (0.85, 1.82) | 1.34 (0.91, 1.99) | 1.46 (0.89, 2.40) | 0.71 (0.48, 1.06) | 0.67 (0.43, 1.06) |
| *P-heterogeneity* | 0.21 | 0∙08 | **0.02** | **0∙01** | 0.83 | 0∙62 |
| *P-trend* | **0.02** | 0.46 | 0.06 | **0.04** | 0.07 | 0.05 |
|  |  |  |  |  |  |  |
| Non-specific febrile symptoms *Q1* | Ref | Ref | Ref | Ref | Ref | Ref |
| *Q2* | 0.83 (0.59, 1.18) | 0.91 (0.63, 1.31) | 0.46 (0.30, 0.69) | 0.67 (0.41, 1.08) | 0.97 (0.66, 1.44) | 0.92 (0.61, 1.40) |
| *Q3* | 0.83 (0.59, 1.17) | 0.84 (0.58, 1.22) | 0.76 (0.51, 1.13) | 1.13 (0.67, 1.92) | 1.36 (0.92, 2.02) | 1.48 (0.90, 2.44) |
| *Q4* | 0.83 (0.55, 1.25) | 0.83 (0.55, 1.24) | 0.81 (0.55, 1.21) | 0.82 (0.53, 1.27) | 1.09 (0.75, 1.60) | 1.12 (0.74, 1.69) |
| *P-heterogeneity* | 1.00 | 0∙89 | **0.01** | 0∙08 | 0.23 | 0∙10 |
| *P-trend* | 0.38 | 0.69 | 0.80 | 0.77 | 0.32 | 0.37 |
|  |  |  |  |  |  |  |
| Home environment *Q1* | Ref | Ref | Ref | Ref | Ref | Ref |
| *Q2* | 1.15 (0.83, 1.61) | 1.05 (0.73, 1.51) | 1.35 (0.90, 2.05) | 1.12 (0.72, 1.76) | 1.14 (0.78, 1.67) | 1.08 (0.72, 1.62) |
| *Q3* | 1.06 (0.76, 1.48) | 0.93 (0.65, 1.33) | 1.72 (1.15, 2.58) | 1.34 (0.86, 2.10) | 1.08 (0.74, 1.59) | 1.06 (0.71, 1.58) |
| *Q4* | 1.22 (0.87, 1.70) | 1.15 (0.81, 1.63) | 1.29 (0.86, 1.93) | 1.05 (0.66, 1.67) | 1.34 (0.93, 1.93) | 1.29 (0.87, 1.92) |
| *P-heterogeneity* | 0.72 | 0∙52 | 0.32 | 0∙51 | 0.53 | 0∙55 |
| *P-trend* | 0.34 | 0.54 | 0.13 | 0.58 | 0.16 | 0.23 |

* Crude odds ratios † Model adjusted further for age using five groups (0-2, 3-5, 6-8, 9-11 and 12-15 years with dummy variables), sex and study region and the six extracted factors (SES, animals, malaria, bed nets, Non-specific febrile symptoms and home factor)

**Supplementary Table 4.** Odds ratios and 95% confidence intervals (CIs) of the association of factors with eBL risk, by country.

|  | **Uganda** | | **Tanzania** | | **Kenya** | |
| --- | --- | --- | --- | --- | --- | --- |
|  | **OR (95% CI)*** | **aOR (95% CI)** † | **OR (95% CI)*** | **aOR (95% CI)** **†** | **OR (95% CI)*** | **aOR (95% CI)** **†** |
| **Malaria infection** |  |  |  |  |  |  |
| Microscopy/Rapid Diagnostic Test (RDT) |  |  |  |  |  |  |
| Negative on both tests | Ref | Ref | Ref | Ref | Ref | Ref |
| Positive on microscopy or RDT | 0.52 (0.46, 0.58) | 0.54 (0.39, 0.74) | 0.40 (0.33, 0.49) | 0.48 (0.26, 0.89) | 0.29 (0.24, 0.34) | 0.30 (0.20, 0.46) |
|  |  |  |  |  |  |  |
| Malaria infection |  |  |  |  |  |  |
| Negative on both tests | Ref | Ref | Ref | Ref | Ref | Ref |
| Positive on RDT, negative on microscopy | 0.62 (0.52, 0.75) | 0.58 (0.34, 0.98) | 0.61 (0.46, 0.82) | 0.83 (0.31, 2.25) | 0.18 (0.13, 0.24) | 0.15 (0.05, 0.45) |
| Positive on microscopy regardless of RDT result | 0.50 (0.44, 0.57) | 0.55 (0.39, 0.78) | 0.40 (0.32, 0.50) | 0.46 (0.22, 0.99) | 0.41 (0.35, 0.49) | 0.44 (0.27, 0.71) |
| *P-trend* | **<0.0001** | **0.0002** | **<0.0001** | **0.03** | **<0.0001** | **0.00003** |
| **Factors** |  |  |  |  |  |  |
| Socioeconomic status *Q1* | Ref | Ref | Ref | Ref | Ref | Ref |
| *Q2* | 1.45 (0.90, 2.33) | 2.27 (1.25, 4.12) | 0.58 (0.33, 1.02) | 0.61 (0.30, 1.23) | 1.50 (0.87, 2.61) | 1.38 (0.73, 2.59) |
| *Q3* | 2.46 (1.66, 3.64) | 3.68 (2.24, 6.04) | 0.74 (0.44, 1.25) | 0.41 (0.20, 0.83) | 2.57 (1.52, 4.34) | 2.92 (1.61, 5.29) |
| *Q4* | 1.67 (1.04, 2.70) | 2.06 (1.16, 3.66) | 1.01 (0.62, 1.64) | 0.79 (0.41, 1.52) | 4.66 (2.87, 7.58) | 3.99 (2.24, 7.11) |
| *P-trend* | **0.003** | **0∙002** | 0.81 | 0.40 | **<0.0001** | **<0.0001** |
|  |  |  |  |  |  |  |
| Animals *Q1* | Ref | Ref | Ref | Ref | Ref | Ref |
| *Q2* | 1.92 (1.10, 3.33) | 3.01 (1.61, 5.63) | 0.85 (0.46, 1.55) | 1.01 (0.49, 2.09) | 0.89 (0.61, 1.31) | 1.11 (0.69, 1.80) |
| *Q3* | 4.24 (2.60, 6.92) | 7.01 (4.03, 12.19) | 1.61 (0.96, 2.70) | 3.88 (1.96, 7.68) | 0.61 (0.39, 0.95) | 0.77 (0.47, 1.26) |
| *Q4* | 6.12 (3.77, 9.93) | 7.05 (3.94, 12.61) | 1.14 (0.66, 1.98) | 3.59 (1.74, 7.43) | 0.56 (0.37, 0.86) | 0.73 (0.44, 1.22) |
| *P-trend* | **<0.0001** | **<0.0001** | 0.22 | **<0.0001** | **0.002** | 0.06 |
|  |  |  |  |  |  |  |
| Inpatient malaria *Q1* | Ref | Ref | Ref | Ref | Ref | Ref |
| *Q2* | 2.18 (1.47, 3.23) | 1.92 (1.21, 3.03) | 1.00 (0.57, 1.76) | 1.08 (0.54, 2.16) | 0.82 (0.51, 1.30) | 1.12 (0.64, 1.96) |
| *Q3* | 1.37 (0.91, 2.06) | 1.26 (0.75, 2.12) | 0.84 (0.46, 1.54) | 1.07 (0.51, 2.22) | 0.69 (0.41, 1.15) | 0.93 (0.51, 1.71) |
| *Q4* | 1.65 (1.12, 2.44) | 1.59 (0.96, 2.64) | 1.54 (0.93, 2.56) | 1.93 (1.01, 3.71) | 2.46 (1.67, 3.64) | 2.29 (1.41, 3.73) |
| *P-trend* | 0.20 | 0.24 | 0.13 | 0.11 | **<0.0001** | **<0.0001** |
|  |  |  |  |  |  |  |
| Outpatient malaria *Q1* | Ref | Ref | Ref | Ref | Ref | Ref |
| *Q2* | 3.40 (1.89, 6.12) | 3.16 (1.63, 6.12) | 2.13 (0.96, 4.73) | 3.78 (1.52, 9.39) | 2.67 (1.65, 4.30) | 2.36 (1.36, 4.09) |
| *Q3* | 5.22 (3.00, 9.07) | 6.78 (3.65, 12.62) | 4.38 (2.15, 8.92) | 7.75 (3.23, 18.63) | 2.37 (1.47, 3.84) | 1.74 (0.99, 3.06) |
| *Q4* | 8.53 (5.00, 14.56) | 10.69 (5.92, 19.31) | 4.27 (2.09, 8.71) | 9.35 (3.87, 22.58) | 2.08 (1.23, 3.52) | 1.85 (1.01, 3.40) |
| *P-trend* | **<0.0001** | **<0.0001** | **<0.0001** | **<0.0001** | **0.03** | 0.20 |
|  |  |  |  |  |  |  |
| Non-specific febrile symptoms *Q1* | Ref | Ref | Ref | Ref | Ref | Ref |
| *Q2* | 1.07 (0.63, 1.84) | 1.53 (0.87, 2.70) | 11.19 (2.60, 48.19) | 9.69 (2.11, 44.43) | 1.45 (0.75, 2.81) | 1.54 (0.75, 3.17) |
| *Q3* | 1.71 (1.02, 2.88) | 2.74 (1.47, 5.12) | 11.28 (2.61, 48.72) | 11.09 (2.38, 51.71) | 1.75 (0.98, 3.13) | 1.88 (0.92, 3.87) |
| *Q4* | 5.81 (3.58, 9.44) | 7.56 (4.35, 13.11) | 39.98 (9.70, 164.79) | 27.42 (6.43, 116.84) | 6.44 (3.82, 10.86) | 5.69 (3.16, 10.23) |
| *P-trend* | **<0.0001** | **<0.0001** | **<0.0001** | **<0.0001** | **<0.0001** | **<0.0001** |
|  |  |  |  |  |  |  |
| Home environment *Q1* | Ref | Ref | Ref | Ref | Ref | Ref |
| *Q2* | 1.27 (0.80, 2.02) | 1.21 (0.70, 2.08) | 1.16 (0.66, 2.03) | 1.66 (0.84, 3.29) | 1.75 (1.09, 2.81) | 1.91 (0.99, 3.66) |
| *Q3* | 2.20 (1.46, 3.31) | 1.96 (1.21, 3.18) | 0.95 (0.54, 1.67) | 1.43 (0.71, 2.89) | 1.32 (0.82, 2.12) | 1.96 (1.13, 3.38) |
| *Q4* | 3.38 (2.27, 5.03) | 3.39 (2.10, 5.46) | 1.22 (0.72, 2.07) | 1.75 (0.84, 3.62) | 1.85 (1.19, 2.87) | 2.20 (1.27, 3.80) |
| *P-trend* | **<0.0001** | **<0.0001** | 0.63 | 0.17 | **0.04** | **0.007** |

* Crude odds ratios † Model further adjusted for age using five groups (0-2, 3-5, 6-8, 9-11 and 12-15 years with dummy variables), sex and study region and the six extracted factors (SES, animals, malaria, bed nets, Non-specific febrile symptoms and home factor)
